# Pressure-Induced Structural Evolution and Negative Compressibility in Hybrid Metal Oxides

**DOI:** 10.1063/4.0000927

**Published:** 2025-10-27

**Authors:** Raúl Torres-Cadena, W. Lakna N. Dayaratne, Hsing-Ta Chen, Evgenii L. Kovrigin, Matthew G. Tucker, Bianca Haberl, Adam Jaffe

**Affiliations:** 1 University of Notre Dame, Notre Dame, IN, USA; 2 Oak Ridge National Laboratory, Oak Ridge, TN, USA

## Abstract

Layered organic-inorganic metal oxides are an underexplored material class that can be isolated in single-crystal and microcrystalline powder form from aqueous, aerobic self-assembly reactions and show promise for implementation in next-generation technologies spanning sensing, optoelectronics, shielding, and energy storage. These compounds interleave (1) two-dimensional metal-oxide layers featuring tunable topologies, compositions, and electronic structures with (2) ordered arrays of molecular species that can direct structure and impart greater chemical functionality. To understand and ultimately control relationships between structural and electronic behaviors, we utilize high pressure as an investigative tool to systematically tune bulk symmetry and to traverse phase boundaries, such as through the formation or severance of bonds. This presentation will detail our crystallographic and spectroscopic analysis of hybrid metal oxides under pressure that have revealed intriguing emergent compression-induced phenomena, including apparent negative compressibility.

